# Study of Usutu virus neuropathogenicity in mice and human cellular models

**DOI:** 10.1371/journal.pntd.0008223

**Published:** 2020-04-23

**Authors:** Marion Clé, Jonathan Barthelemy, Caroline Desmetz, Vincent Foulongne, Lina Lapeyre, Karine Bolloré, Edouard Tuaillon, Nejla Erkilic, Vasiliki Kalatzis, Sylvie Lecollinet, Cécile Beck, Nelly Pirot, Yaël Glasson, Fabien Gosselet, Maria Teresa Alvarez Martinez, Philippe Van de Perre, Sara Salinas, Yannick Simonin

**Affiliations:** 1 Pathogenesis and Control of Chronic Infections, University of Montpellier, INSERM, EFS, Montpellier, France; 2 BioCommunication en CardioMétabolique (BC2M), University of Montpellier, Montpellier, France; 3 Inserm U1051, Institute for Neurosciences of Montpellier. University of Montpellier, Montpellier, France; 4 UPE, Anses Animal Health Laboratory, INRA, Anses, ENVA, Maisons-Alfort, France; 5 BCM, University of Montpellier, CNRS, INSERM, Montpellier, France; 6 IRCM, University of Montpellier, ICM INSERM, Montpellier, France; 7 University of Artois, Blood-Brain Barrier Laboratory (BBB Lab), France; 8 RAM-ECE, BioCampus Montpellier, CNRS, INSERM, University of Montpellier, Montpellier France; 9 Centre Hospitalier Universitaire de Montpellier, Montpellier, France; Center for Disease Control and Prevention, UNITED STATES

## Abstract

Usutu virus (USUV), an African mosquito-borne flavivirus closely related to West Nile virus, was first isolated in South Africa in 1959. USUV emerged in Europe two decades ago, causing notably massive mortality in Eurasian blackbirds. USUV is attracting increasing attention due to its potential for emergence and its rapid spread in Europe in recent years. Although mainly asymptomatic or responsible for mild clinical signs, USUV was recently described as being associated with neurological disorders in humans such as encephalitis and meningoencephalitis, highlighting the potential health threat posed by the virus. Despite this, USUV pathogenesis remains largely unexplored. The aim of this study was to evaluate USUV neuropathogenicity using *in vivo* and *in vitro* approaches. Our results indicate that USUV efficiently replicates in the murine central nervous system. Replication in the spinal cord and brain is associated with recruitment of inflammatory cells and the release of inflammatory molecules as well as induction of antiviral-responses without major modulation of blood-brain barrier integrity. Endothelial cells integrity is also maintained in a human model of the blood-brain barrier despite USUV replication and release of pro-inflammatory cytokines. Furthermore, USUV-inoculated mice developed major ocular defects associated with inflammation. Moreover, USUV efficiently replicates in human retinal pigment epithelium. Our results will help to better characterize the physiopathology related to USUV infection in order to anticipate the potential threat of USUV emergence.

## Author summary

Number of emerging arboviruses involved in human infections has increased considerably in the past years. Among them, Usutu virus (USUV) is an African mosquito-borne virus first isolated in South Africa that recently emerged. USUV infection in humans is considered to be most often asymptomatic or to cause mild clinical signs. Nonetheless, increased cases of neurological complications such as encephalitis or meningoencephalitis have been reported in Europe but the mechanisms behind this neuropathogenesis remain largely unclear. In this study we showed that USUV can infect efficiently several organs and cells of the central nervous system associated with a drastic inflammation and various deleterious effects. Our results contribute to the characterization of the neurotropism related to USUV infection.

## Introduction

Among emerging viruses, Usutu virus (USUV) has recently attracted the attention of the scientific community due to its extensive spread in Europe, which has grown in recent years. USUV is an African mosquito-borne virus that was first identified in 1959 in South Africa [1]. Like West Nile virus (WNV), with which it shares many common features, it belongs to the Japanese encephalitis (JEV) serocomplex in the *Flavivirus* genus (*Flaviviridae* family) [2,3]. USUV is an enveloped virus of approximately 40–60 nm in diameter. Its genome is a single-stranded RNA of positive polarity comprised of 11,064 nucleotides harboring a 5' N7-methylguanosine-triphosphate cap but lacking a polyA tail at the 3’ end [4]. The genome has a single open reading frame coding for a polyprotein of 3,434 amino acids that, after cleavage, gives rise to three structural proteins (capsid C, premembrane prM, and envelope E) and eight non-structural proteins (NS1/NS1’, NS2a, NS2b, NS3, NS4a, 2K, NS4b, and NS5) [2]. Phylogenetic studies have shown that USUV strains can be divided into 8 lineages: 3 African and 5 European [5]. The originally isolated USUV strain SAAR 1776 belongs to African 2 lineage that has been known to be circulating in Europe for several years and was recently identified in a clinical case of a *frigore* facial paralysis in France [6,7]. Phylogenetic analyses suggest that at least three USUV introductions have occurred in Europe along the bird migratory routes from Africa [6]. The natural life cycle of USUV is very similar to that of WNV. The virus is maintained through an enzootic cycle involving mainly birds of the orders Passeriformes and Strigiformes as amplifying hosts and ornithophilic mosquitoes, like the common *Culex pipens* which is widespread in Europe, as vectors [8]. Major central nervous system (CNS) disorders such as prostration, disorientation, and ataxia have been reported in USUV-infected birds, leading to death. Necrotic areas and inflammatory infiltrates composed of lymphoid and histiocytic cells also have been reported in the brain of infected birds [9]. Mammals, including wild boars, were described as accidental or dead-end hosts [10–12]. USUV has also been detected in bats and in different rodents and shrew species, suggesting the possibility of the existence of non-avian reservoirs [13,14]. Up to 2015, USUV infection has been reported in mosquitoes, birds, and horses in a dozen of European countries. During the summer of 2016, major USUV epizootics affecting avifauna were evidenced in Northern Europe, and in 2018, USUV spread rapidly in Western Europe, associated with a large WNV epidemic [8,15–18].

In humans, USUV infection was first described in Africa (Central African Republic and Burkina Faso in 1981 and 2004, respectively), with mild clinical signs such as fever and skin rash reported [12]. In Europe, the recent epizootics were also accompanied by several descriptions of human neurological disorders, including facial paralysis, encephalitis, meningitis and meningoencephalitis, in immunocompromised and immunocompetent patients, representing to date around 30 cases [7,18–25]. Moreover, molecular and serologic evidence of USUV infection in Italian, German, Austrian and Dutch blood donors indicates that the virus is also circulating silently among asymptomatic humans in Europe and could potentially be a concern for blood transfusions [26–29]. Very little is known about USUV pathogenicity. USUV, like many flaviviruses, is neurotropic. It can infect murine and human neuronal cells *in vitro*, leading to apoptosis or proliferation arrest [30]. Adult wild mice do not appear susceptible to USUV infection [10]. In contrast, mice lacking the interferon type 1 receptor (*Ifnar1*^*-/-*^) were highly sensitive to USUV neuroinvasive infection, as has been described for other flaviviruses [31]. Experimental infections of one-week-old immunocompetent-Swiss and *Ifnar1*^*-/-*^ mice by intraperitoneal injection reproduced neurological signs such as depression, paraplegia, paralysis and coma [10,32].

In this study, we attempted to better characterize USUV neuropathogenicity using mice and human cellular models. We demonstrated that USUV efficiently replicates in the spinal cord and brain of mice associated with infiltration of immune cells and strong induction of pro-inflammatory cytokines. We also showed that USUV can infect a human model of the blood-brain barrier (BBB) without altering its integrity. Moreover, USUV-inoculated mice developed drastic ocular defects. To study the ability of USUV to replicate in the human retina, we infected confluent induced pluripotent stem cell (iPSC)-derived retinal pigment epithelium (RPE) cultured on transwell filters [33]. We observed USUV replication and cytokine induction that was not associated with a loss of epithelium integrity.

## Results

### USUV induces weight loss, motor impairment and mortality in mice

**Fig 1:**
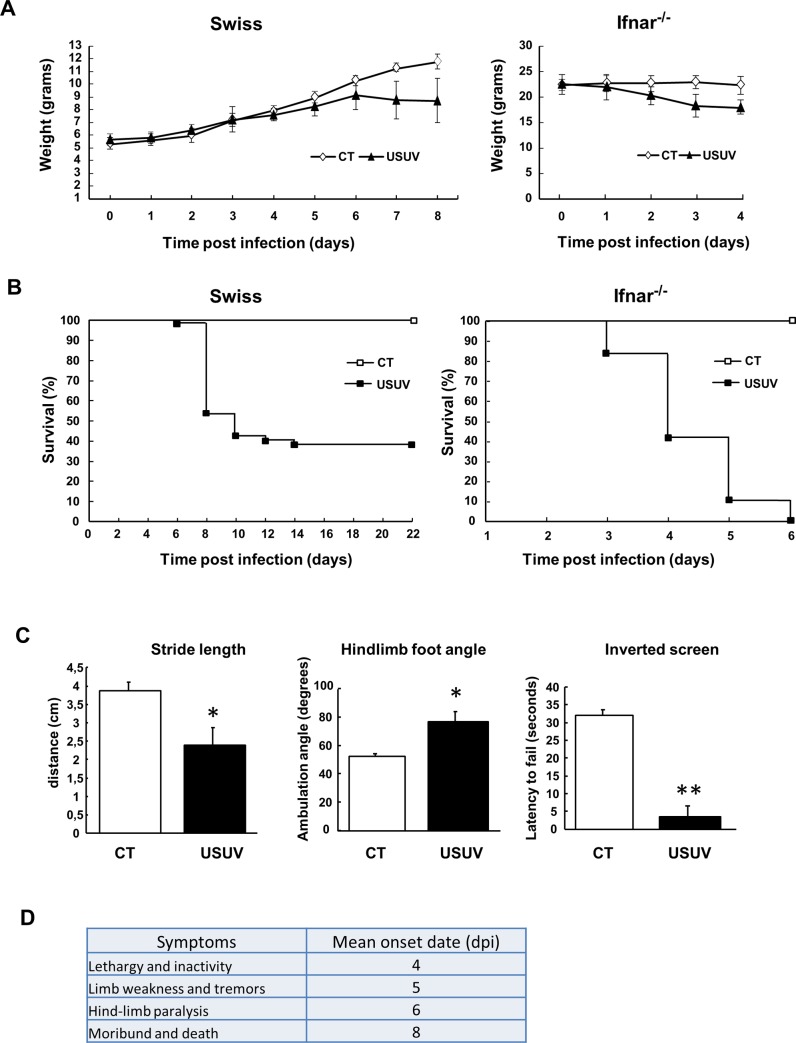
Pathogenic manifestations of USUV infection in suckling immunocompetent and Ifnar^−/−^ mice. (A) Weight tracking and (B) survival rates of neonatal Swiss mice (6 days old) and Ifnar^−/−^ mice (80 days old) intraperitoneally inoculated with 10^4^ TCID50/mouse of USUV. CT = control mice. Infection by USUV is lethal at 60% for suckling Swiss mice and at 100% for Ifnar^−/−^ mice. Infected mice lose weight over time. (C) Left panel. Footprint assay of gait abnormalities in control and USUV-infected suckling mice. The stride length and hindlimb foot angle were measured using hind paws marked with blue ink. USUV-inoculated animals have a greater foot angle and a reduced stride length compared to non-infected mice, indicating motor defects. Right panel. Endurance measured by the inverted screen test shows that infected mice exhibited significantly shorter latency to fall. (D) Signs, morbidity rate, and onset date of USUV-infected suckling mice. Lethargy appears around 4 dpi and mice die on average at 8 days. Mice were monitored daily until clinical signs of disease were displayed and then were euthanized. n = 20 for each group. * p < 0.05, ** p <0.01.

To better characterize USUV neuroinvasiveness, we first assessed the susceptibility of mice to USUV infection. Many *Flaviviruses*, such as dengue virus (DENV) and Zika virus (ZIKV), classically do not effectively infect immunocompetent mice, likely due to an absence of species-specific immune evasion mechanisms [34,35]. For example, NS5 protein from ZIKV antagonizes human but not mouse STAT2, which transmits signals downstream of IFNAR1, a component of the type I IFN receptor [36]. To overcome this limitation, it is necessary to use either mice deficient in the interferon receptor (*Ifnar* ^-/-^) gene or immunocompetent neonatal mice [34]. Previous reports of USUV-associated neuropathology in mice have shown that these animal models are pertinent to study USUV neuropathogenicity [31,32]. *Ifnar*^-/-^ mice (12 weeks old) and suckling immunocompetent Swiss mice (6 days old) were intraperitoneally (i.p.) inoculated and weight loss and motor defects were monitored until the end of the experiment. Infected animals progressively lost weight or failed to gain weight in both mice models (Fig 1A). In infected mice, the first signs (lethargy and inactivity) appeared 4 days post infection (dpi) and mean time to death was 8 dpi (Fig 1B–1D). All infected *Ifnar*^-/-^ mice died 6 dpi whereas suckling mice had 60% mortality at 14 dpi in accordance with a previous study (Fig 1B) [10]. We subsequently decided to focus primarily on studying the effect of USUV in immunocompetent suckling mice. Motor functions were examined at 6 dpi in neonatal mice using basic established motoric tests. Footprint assays, used to determined gait ataxia, showed characteristics of gait abnormalities in infected mice, such as a reduced stride length and increased hindlimb foot angle (Fig 1C). Endurance strength, measured through an inverted screen test, was significantly impaired in symptomatic USUV mice (around 60%), which exhibited significantly shorter latency to fall (Fig 1C). These results show that drastic motor dysfunction in USUV-infected mice appears quickly after infection.

### USUV replicates in multiple organs and tissues of the infected neonates but mainly in the nervous system

**Fig 2:**
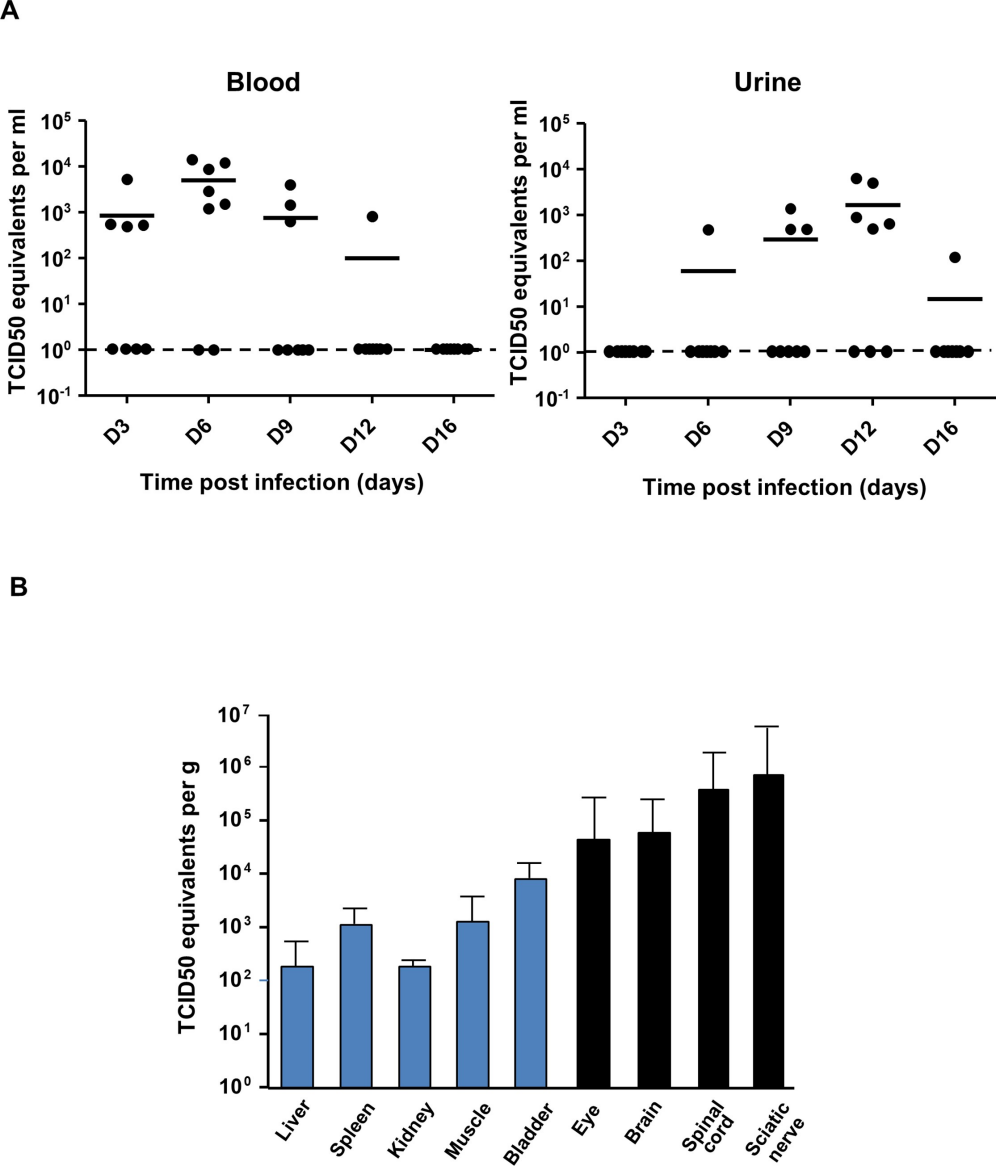
USUV infects multiple organs of neonatal mice but target mainly the CNS. 6-day-old Swiss mice were infected intraperitoneally with USUV at 10^4^ TCID50/mouse. Viral burden was measured by RT-qPCR assay and indicated by TCID50 equivalent per gram. (A) Viral burden in blood and urine at several times post injection. Viral RNA appears earlier in blood (3 dpi) than in urine (6 dpi) and disappears earlier. (B) Viral burden in several organs including CNS at 6 dpi. Viral genome was detected in liver, spleen, hindlimb muscle, kidney, and bladder. The higher amount of viral genome was identified in nervous tissues (black bars). Lower limit of detection is of 50 TCID50 equivalent per gram or per ml.

To better characterize USUV tropism, mice were dissected following euthanasia to isolate several organs and tissues. RT-qPCR targeting the NS5 gene of USUV was performed to quantify viral genome of USUV-infected mice as compared with the PBS-treated mice [37]. First, viral load was determined in blood and urine. Interestingly, viral RNA appears earlier in blood (3 dpi) than in urine (6 dpi) but also disappears earlier as described for several other flavivirus (Fig 2A). The late presentation of the viral RNA in the urine, which is predominantly detected at 12 dpi, suggests that urination could be a route for the excretion of the virus. Viral genome was then identified by RT-qPCR at 6 dpi in all other tested tissues: liver, spleen, hindlimb muscle, kidney, and bladder (Fig 2B). Interestingly, the highest amounts of viral genome were detected in nervous tissues such as eyes (including optic nerve), brain, spinal cord and sciatic nerves (Fig 2B, black bars).

Therefore, our results suggest that USUV infects and replicates in various tissues and organs, with a greater tropism for the central and peripheral nervous system.

### USUV induces massive brain inflammation

**Fig 3:**
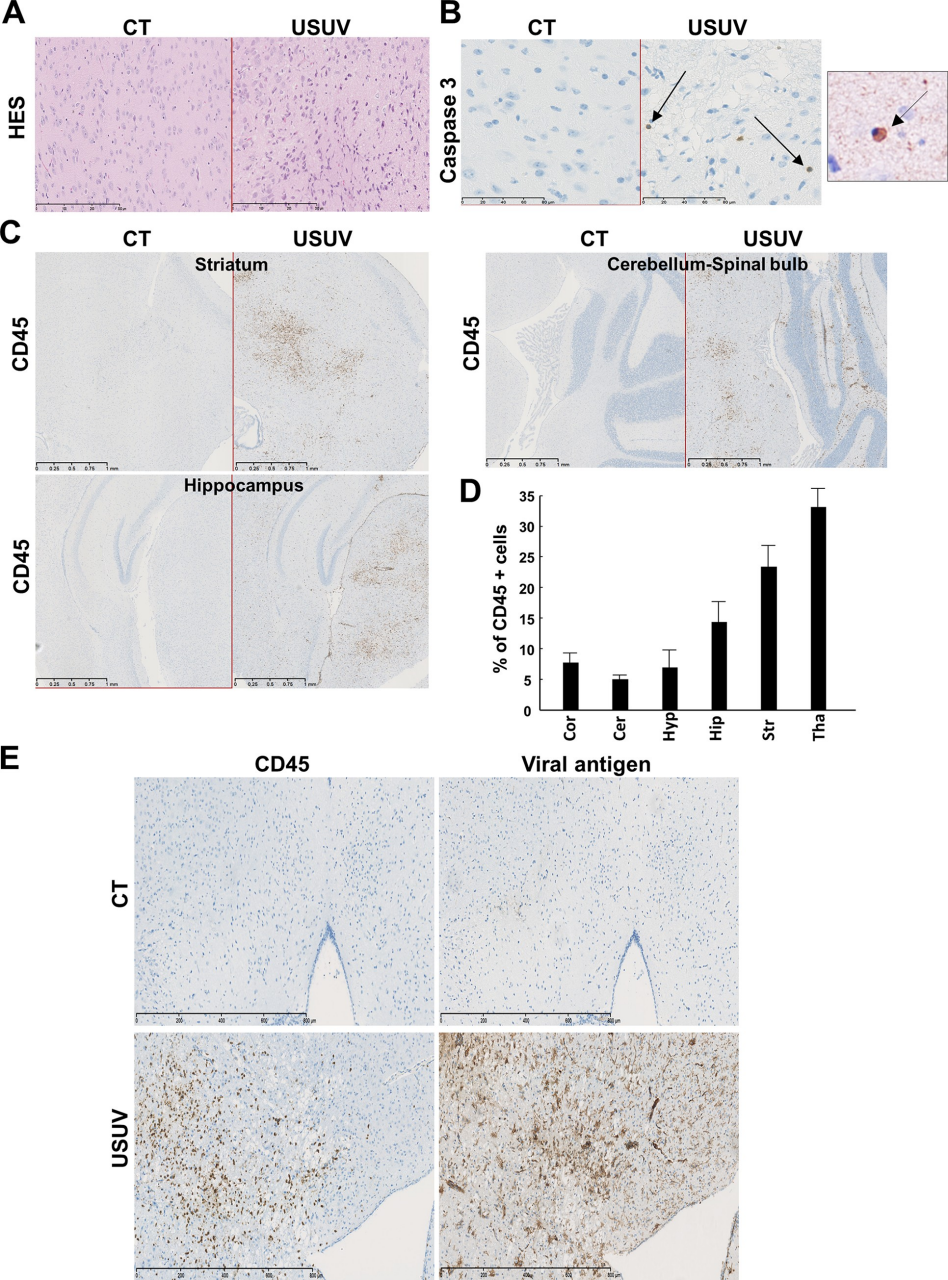
USUV induces apoptosis and cellular infiltration in the mouse brain. **(**A) Transverse sections of the brain (thalamus) of USUV-infected and control (mock, PBS injected) mice at 6 dpi stained with haematoxylin/eosin/saffron. We observe cellular infiltration and cell shrinkage. CT = control mice. (B) Some cells present nuclear condensation and caspase 3 staining after immunohistochemistry, suggesting apoptotic process in USUV-infected mice. Right panel: zoom on apoptotic cell. Arrows indicate apoptotic cells. (C) Immunohistochemical CD45 staining (associated with luxol blue) showing massive inflammatory infiltrates in multiple areas of the infected brain. (D) Quantification of CD45 positive cells in different brain area. Cor: Cortex, Cer: Cerebellum, Hyp: Hypothalamus, Hip: Hippocampus, Str: Striatum, Tha: Thalamus. (E) Immunohistochemical staining of viral antigen and CD45. The majority of infected cells are not inflammatory cells.

**Fig 4:**
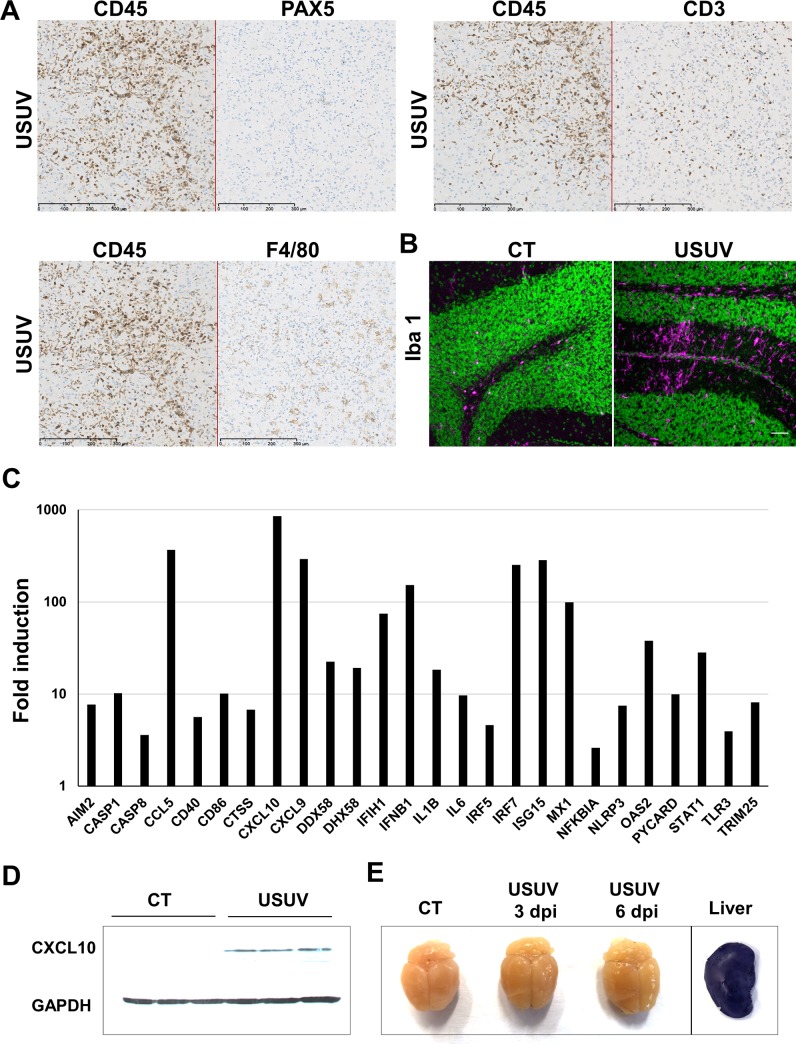
USUV induces massive brain inflammation. Histological sections of neonatal brain mice infected by USUV (6 dpi). (A) Immunohistochemical staining of B-cells (PAX 5), T-cells (CD3) and macrophages (F4/F80) showing T-cells and macrophages infiltration. (B) Immunofluorescence staining (Iba1 in pink, nucleus in green) shows microglia recruitment/activation in USUV-infected brain contrary to control mice (CT). (C) RT-qPCR analysis using a specific inflammatory cytokines and receptors PCR array of mRNA collected at 6 dpi from brain of control and infected mice. Fold regulation of statistically significant genes normalized to CT are indicated for 3 mice of each group. *p* <0.05 (p value in S3A Fig). Scale bar = 50μm. (D) CXCL10 protein level in control and infected mice. (E) Representative picture of dissected brain from EB-injected mock- and USUV- infected mice at 3 and 6 dpi showing no blue labelling. Liver is used as positive control of EB staining.

As suggested by previous studies, as well as our RT-qPCR results (Fig 2B) and the signs observed in mice (such as limb weakness and hind-limb paralysis), USUV replicates and damages CNS tissues to cause functional abnormalities [10,32]. To better characterize the profile of brain infection, we performed immunofluorescence and immunohistochemistry (IHC) assay in different areas of the CNS in 12-day-old mice (6 dpi). First, brain sections were stained by HES (hematoxylin eosin saffron) to analyze the general histomorphology. We observed massive infiltrating cells (corresponding to 28% of total cells), cell shrinkage and some cells (6,2%) with nuclear condensation associated with caspase 3 staining, suggesting a limited apoptotic process (Fig 3A and 3B). Massive inflammatory infiltrates in multiple areas of the brain, mainly in striatum and thalamus, were highlighted by CD45 staining which recognize all lymphoid cells (Fig 3C and 3D). USUV-staining showed direct infection of the brain that is not correlated with the distribution of inflammatory cells (Fig 3E). Among these inflammatory cells, we detected infiltration of T cells (CD3 staining), macrophage infiltration (F4/F80 staining), but no significant B cell infiltration (PAX 5 staining) (Fig 4A, S1 Fig). Activated microglia are a major source of cytokines/chemokines induction within the inflamed brain. To determine if USUV replication and cell death induction correlated with microglia cell recruitment/activation, we stained brain sections for Iba1 (Ionized calcium binding adapter molecule 1), which is upregulated in activated microglia cells. We observed an increase in Iba1 staining in the brains of diseased mice as compared to control mice (Fig 4B). To determine whether this recruitment of inflammatory cells is correlated with overexpression of inflammatory cytokines and induction of anti-viral responses, we used a PCR array to analyze inflammatory cytokines and receptors (Fig 4C) [38]. The gene expression of some key cytokines and chemokines appeared to be strongly increased in USUV-infected brains. Notably, we observed upregulated mRNA expression levels of components of interferon (IFN) signaling such as *IFNβ*, but also interferon regulatory transcription factor (IRF) family such as *IRF5* and *IRF7*, *IFIH1* (interferon induced with helicase C domain 1) and several interferon-stimulated genes (ISGs): *OAS2* (2'-5'-oligoadenylate synthetase 2), *MX1* (MX dynamin-like GTPase 1) and *ISG15* (Interferon stimulated gene 15). It is notable that the main components of inflammasome, such as *NLRP3* (NOD-like receptor family, pyrin domain containing 3), *IL-1β* (interleukine 1 beta), *AIM2* (Absent In Melanoma 2), *CASP1* (caspase 1), *CASP8* (caspase 8) and *PYCARD*, are also overexpressed in USUV-infected mice. This suggests that this complex, involved in innate immunity, is activated following USUV infection as has been previously shown for other flaviviruses like WNV [39,40]. Several chemokines, such as *CXCL10*, *CXCL9* and *CCL5*, were also found highly upregulated in the brain of USUV-infected mice (Fig 4C). These cytokines are known to be increased within the CNS during arboviral encephalitis and are highly induced during JEV, WNV, TBEV (Tick borne encephalitis virus), SFV (Semliki Forest virus), and SINV (Sindbis virus) infection [41]. *CXCL10* has been reported to induce neuron apoptosis or direct damage in neuronal cells [42]. In our experiment, *CXCL10* mRNA is upregulated up to 1000 times the normal level in the brains of USUV-diseased mice (Fig 4C) and *CXCL10* protein level appears also up-regulated in infected mice (Fig 4D). Interestingly, CCL5 protein was also found to be up-regulated in the blood of Swiss and *Ifnar*^-/-^ USUV-infected mice (S2 Fig). Moreover, we observed the upregulation of one of the main inflammatory cytokines, *IL6*, and increased expression of *RIG-I* (DDX58), *LGP2* (DHX58) and *TLR3*, pattern-recognition receptors (PRRs) activated by viral dsRNA during viral replication (Fig 4C, S3 Fig) [43].

Numerous studies have shown that flaviviruses can reach the CNS by crossing the BBB, with or without barrier breakdown, using several pathways, including direct infection of brain microvascular endothelial cells, transcytosis or through infected-immune cells (Trojan-horse mechanism) [44]. To determine if USUV infection is associated with loss of BBB integrity, we infected adult mice (*Ifnar*^*-/-*^) that have fully formed BBB. After infection, animals were subjected to Evans blue (EB, a colorant used to monitor BBB integrity) by intraperitoneal-injection at 3 dpi (as we did not detect viral RNA in brain until 3 dpi) and 6 dpi. There was no blue labelling in brain suggesting no major BBB disturbance after USUV infection while other organs, like the liver, were blue (Fig 4E).

Altogether our observations suggest that USUV induces a strong antiviral-response in the CNS, which is associated with inflammation and highlighted by the recruitment of inflammatory cells and the mRNA upregulation of pro-inflammatory cytokines and chemokines as well as of inflammasome components.

### USUV replicates in brain-like endothelial cells of an *in vitro* human BBB model and upregulates inflammatory cytokines and chemokines

**Fig 5:**
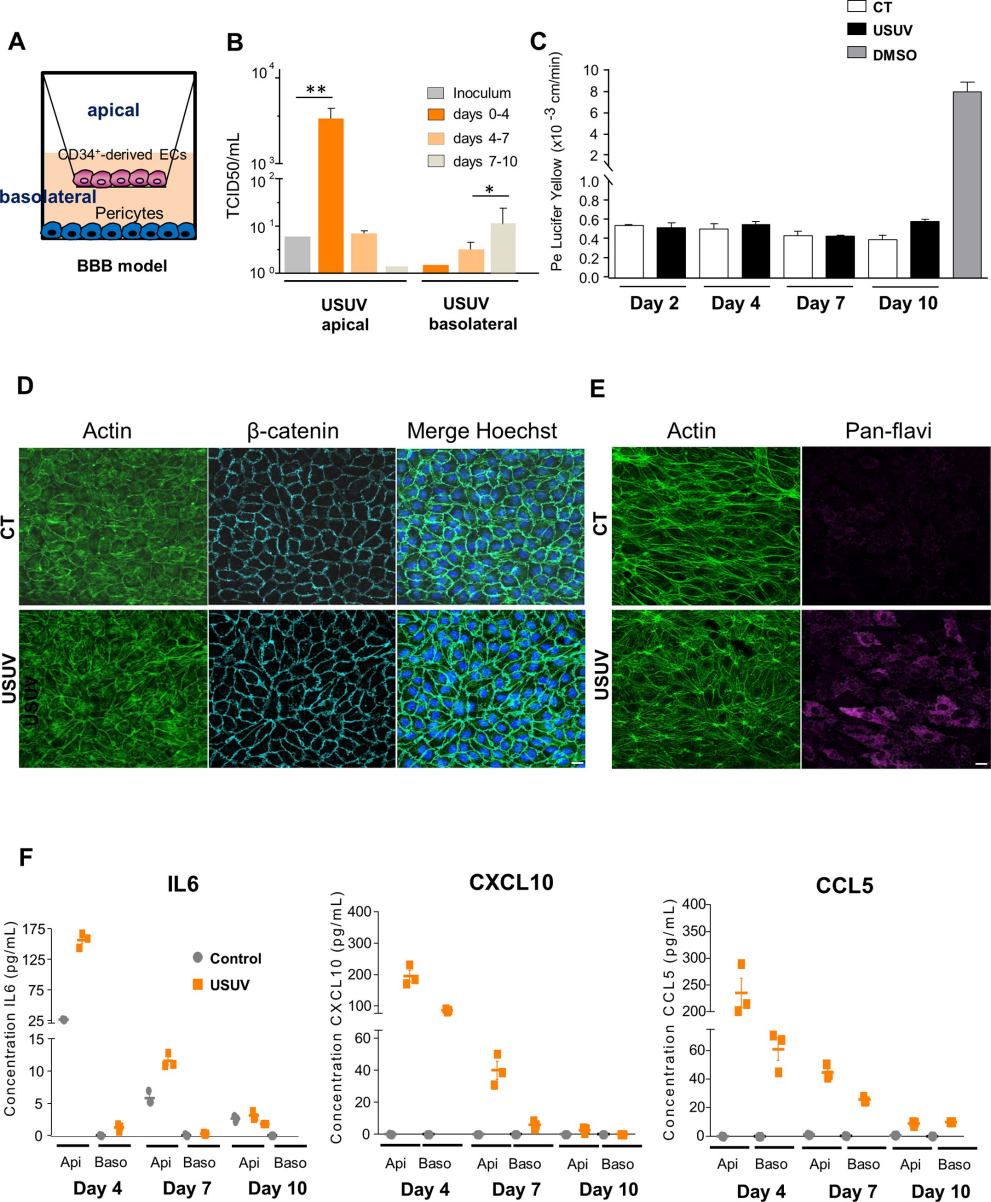
USUV replicates in a human model of the Blood-Brain Barrier without alteration of endothelium integrity. (A) Human *in vitro* BBB model that consists in CD34+ blood cord-derived endothelial cells (CD34+-EC) that have been further differentiated and cultured in transwell filters placed on top of bovine pericytes. (B) BBB model was infected with USUV at MOI 0.1. Viral titers from inoculum, apical and basolateral compartments are indicated at 4 dpi, 7 dpi and 10 dpi. (*p<0.05, **p<0.01). (C) Permeability (Pe) of the Lucifer Yellow (LY) paracellular marker was measured in mock (control, CT)- or USUV-infected BBB models at 2 dpi, 4 dpi, 7 dpi and 10 dpi. Positive control of the opening of BBB: endothelial cells were incubated with 10% dimethylsulfoxide (DMSO) one hour before permeability experiment. Results are expressed as mean ± SEM of 3 independent experiments. (D) Immunofluorescence of actin and β-catenin staining showing no major perturbation of endothelium architecture at 10 dpi. Scale bar = 10μm. (E) Immunofluorescence of actin and pan-flavivirus staining showing infection of endothelium cells. (F) ELISA analyses of IL6, CXCL10 and CCL5 concentrations in the supernatants from apical (api) and basolateral (baso) compartments of mock- or USUV-infected BBB model at 4 dpi, 7 dpi and 10 dpi.

Our *in vivo* results suggest that USUV can cross the BBB without major barrier breakdown and may lead by this mechanism to encephalitis and/or meningoencephalitis *via* inflammatory response. Moreover, we previously shown that USUV, like for other flaviviruses, can infect human neuronal precursors and astrocytes, leading to death by apoptosis or arrest of proliferation, respectively [45]. As arbovirus infection in mice might be species-specific, we determined whether our observations had relevance to human tissues. To address this issue, we sought to determine whether USUV could directly infect the human BBB and perturb its integrity. We used an innovative human *in vitro* BBB model that recapitulates the main characteristics of the endothelial barrier observed *in vivo*. Briefly, CD34^+^ cord blood-derived hematopoietic stem cells were allowed to differentiate on culture inserts in contact with pericytes for 6–7 days to acquire BBB characteristics (Fig 5A) [46]. These brain-like endothelial cells (hBLECs) express tight junctions and transporters typically observed in brain endothelium [46] and allow to study drugs or cells passage across the BBB [47,48]. hBLECs were infected by USUV on the apical side at the multiplicity of infection (MOI) of 0.1. At 4, 7 and 10 dpi supernatants were collected in the apical and basolateral sides and cells were fixed to analyze endothelium integrity. Viral replication was determined by tissue culture infective dose 50% (TCID50). Fig 5B shows viral titers in apical sides (corresponding to blood vessel lumens) at 4 dpi and to a lesser extent at 7 dpi (corresponding to the production between 4 to 7 days, as medium need to be changed at 4 dpi), whereas no efficient production was detected at late time (7 to 10 dpi production). Weak viral production was detected later (at 7 dpi) in the basolateral sides (which would correspond to the parenchyma side) and increased at 10 dpi. We used the clearance of the small paracellular integrity marker, Lucifer Yellow, to evaluate the endothelial permeability coefficient [46]. Endothelium integrity did not appear significantly altered at the studied times (until 10 dpi) as the control and infected endothelium displayed a permeability coefficient of ~0.5x10^-3^ cm/min, consistent with “tight” BBB endothelia (inferior to 1.5). This result was confirmed by β-catenin and actin immunofluorescence at 10 dpi to assess endothelium architecture (Fig 5C and 5D). No significant cytopathic effects (CPE) were observed despite infection of endothelium cells (Fig 5E).

Using ELISA assays, we next measured the concentrations in apical and basolateral sides of CXCL10, CCL5, and IL6, chemokines and cytokines that we previously showed as upregulated in USUV-infected mouse brains. Following USUV infection, we showed secretion of CXCL10, CCL5, and IL6 in apical compartments from 4 to 10 dpi, but the secretion level decreased with time. CXCL10 and CCL5 were also secreted at the basolateral side (Fig 5F).

Altogether, our results suggest that USUV may directly infect the BBB from the luminal side. USUV infectious particles are released from apical and basolateral compartments of the endothelium and could therefore reach the CNS *via* this route. Moreover, productive infection does not significantly alter the BBB integrity as the endothelium remains impermeable and no architectural disturbance was observed. Otherwise, chemokines such as CXCL10 or CCL5 are secreted from both sides in infected hBLECs, potentially leading to the recruitment of inflammatory cells to promote the massive inflammation observed *in vivo*.

### USUV induces spinal cord inflammation

**Fig 6:**
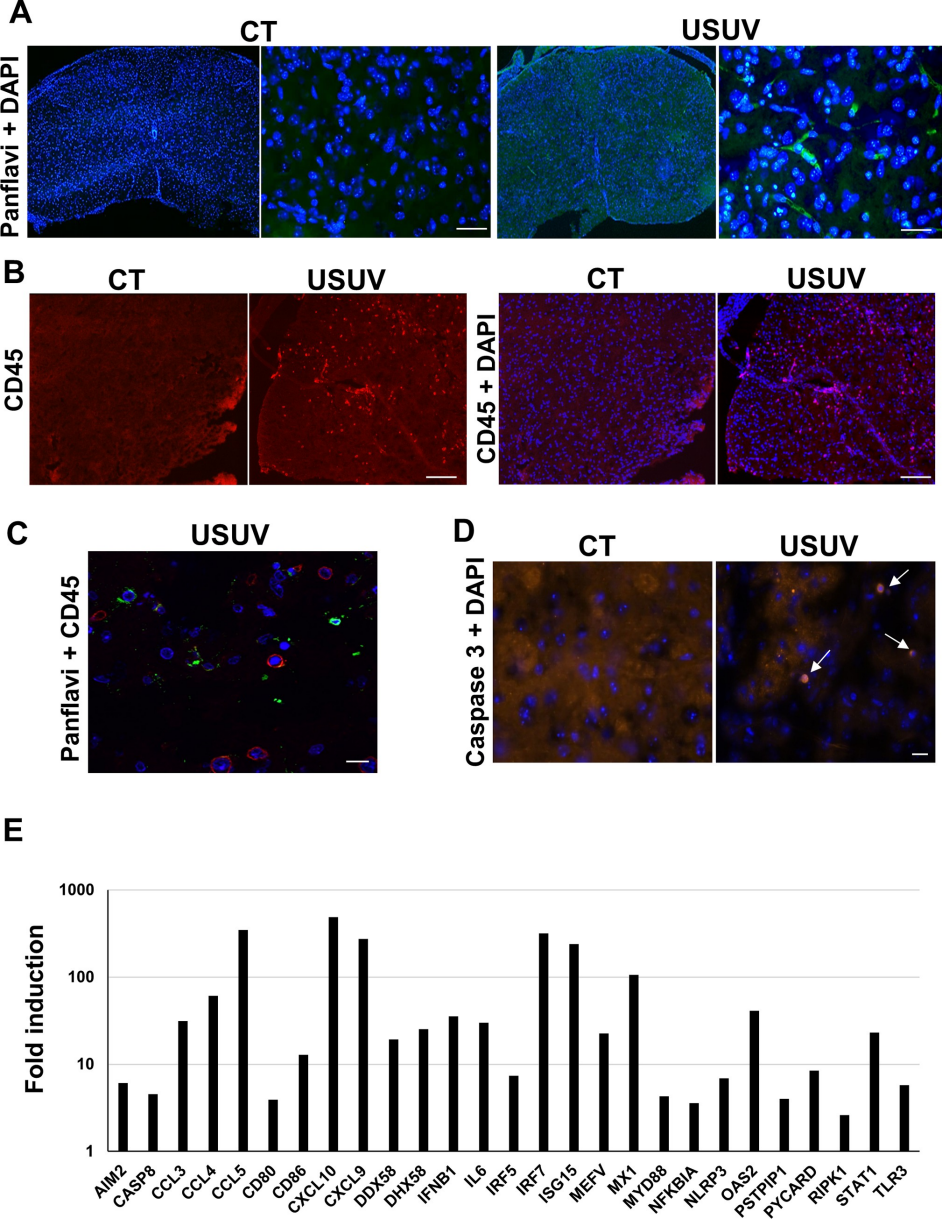
USUV induces spinal cord inflammation. (A) Immunofluoresence of control (CT) and USUV-infected spinal cords of Swiss mice at 6 dpi. The virus is labeled using a pan-flavivirus antibody (green) and nuclei by DAPI (blue). Scale bar = 20 μm. (B) Immunofluoresence CD45 staining showing inflammatory infiltrates in infected-spinal cord (C) not associated with USUV staining (adjacent sections). Scale bar = 15 μm in panel B and 50 μm in panel C. (D) Immunofluoresence caspase 3 staining showing apoptosis in infected-spinal cord. Arrows indicate apoptotic cells. Scale bar = 20 μm. (E) RT-qPCR analysis using a specific inflammatory cytokines and receptors PCR array of mRNA collected at 6 dpi from the spinal cord of control and infected mice. Fold regulation of statistically significant genes normalized to CT are indicated for 3 mice in each group. *p* <0.05 (p value in S3B Fig).

As shown in Fig 2B, a high viral RNA amount was found in the spinal cord of infected mice. We also observed infected cells by immunofluorescence (Fig 6A). This is why we decided to explore the inflammation profile in the spinal cord and to compare it with the one observed for infected brains. First, CD45 staining indicated recruitment of uninfected inflammatory cells in USUV spinal cords (Fig 6B and 6C). We also detected apoptosis (10.1%) in the spinal cord of infected-animals (Fig 6D). Interestingly, the inflammation profile studied by PCR-array was very similar in the spinal cords and brains of USUV-infected mice. We detected in particular overexpression of the same chemokines (*CXCL10*, *CXCL9*, *CCL5*), as well as of spinal cord-specifically upregulated *CCL3* and *CCL4* chemokines. Antiviral response was also overexpressed (*IFNβ*, *OAS2*, *MX1*, *ISG15*, *IRF5*, *IRF7*, *…*) as well as inflammasome components (*NLRP3*, *CASP8*), and we found the same PRRs overexpressed in the spinal cord as in the brain (*RIG-I*, *LGP2* and *TLR3)* (Fig 6E, S4 Fig).

### USUV induces inflammatory responses in the eye and efficiently replicates in human retinal pigment epithelium

**Fig 7:**
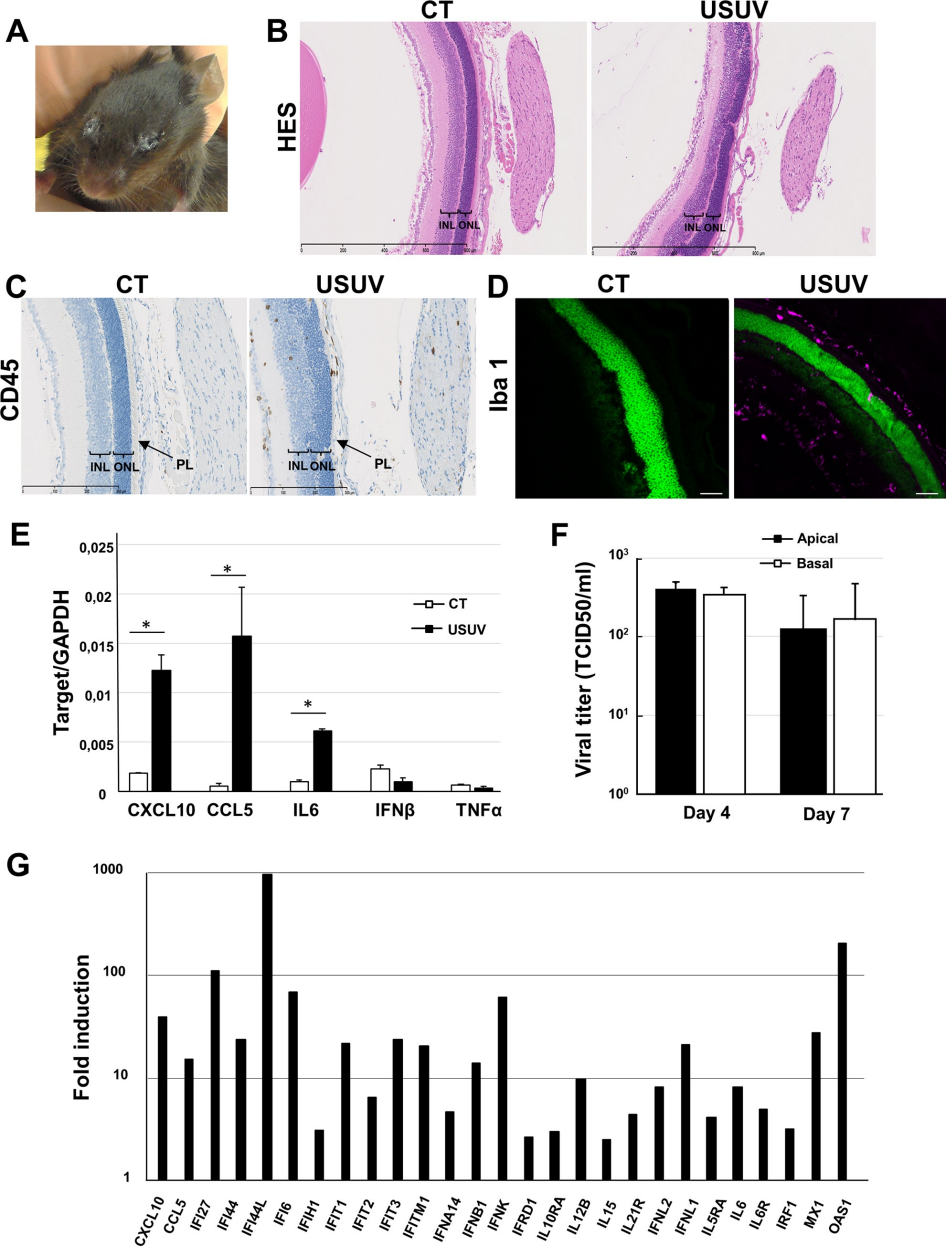
USUV induces strong inflammatory responses in mouse eyes and efficiently replicates in human retinal pigment epithelium. (A) Representative photograph demonstrating ocular pathology and exudate in Ifnar^−/−^ mice inoculated intraperitoneally with USUV at 10^4^ TCID50/mouse. (B) Histological sections of uninfected (CT) or USUV-infected (6 dpi) neonatal mouse eye stained with haematoxylin/eosin/saffron and (C) immunohistochemical CD45 staining showing inflammatory infiltrates in neonatal eyes. Disruption of both inner and outer retinal structure and loss of photoreceptors layer were observed. INL: inner nuclear layer. ONL: outer nuclear layer. PL: photoreceptors layer. (D) Immunofluorescence staining shows microglia recruitment/activation in eyes of infected mice (Iba1: purple, nuclei: false colored green). Scale bar = 50μm. (E) qRT-PCR analysis of CXCL10, CCL5, IL6, IFNα and TNFα mRNA collected at 6 dpi from eyes of control and infected mice. *p <0 .05. (F) IPSC-derived-RPE were infected with USUV at MOI 0.2. Viral titers from apical and basolateral compartments of RPE grown on cell culture inserts were measured at 4 dpi and 7 dpi. Results are expressed as mean ± SEM of 3 independent experiments. (G) Fold regulation of statistically significant genes modulated at 2 dpi normalized to CT in USUV-infected RPE by qRT-PCR analysis using a specific interferons and receptors PCR array. Results are expressed as means of the fold regulation for 3 independent experiments and analyzed using a Mann-Whitney test, *p* <0.05 (p value in S4 Fig).

As an extension of the CNS, the eye displays similarities to the brain and spinal cord in terms of anatomy, functionality, response to injury, and inflammation [49]. Although eyes are sequestered from the systemic circulation, numerous viruses, among them arboviruses can still reach this organ and cause inflammation and pathologies [50]. Several flaviviruses, including DENV, ZIKV and WNV, have been described as triggering ophthalmic damage, including retinopathy [51]. Using a RT-qPCR approach, we observed that USUV could replicate in the eyes of neonatal mice (4x10^4^ TCID50 eq per g, Fig 2B) and *Ifnar*^*-/-*^ infected mice (10^5^ TCID50 eq per g). This replication was associated with some ocular defects, including severe conjunctivitis in *Ifnar*^*-/-*^ mice with extraocular exudate, as shown in Fig 7A. Similarly, USUV-infected neonatal mice frequently presented closed eyes. Histological analyses by HES staining showed disruption of both inner and outer retinal structure and a loss of photoreceptors layer (Fig 7B and 7C). Histological analysis revealed otherwise no apoptotic cells after staining with caspase 3 antibody (S5 Fig), whereas CD45 staining revealed infiltration of inflammatory cells in all layers of the posterior compartments, mainly in retinal ganglion cell layer and the inner retina, but also in the optic nerve and in the iris of the anterior eye, suggesting neuroretinitis and uveitis (Fig 7C). As microglial cells are the major population of immune cells in quiescent retina, and are important for the maintenance of retinal homeostasis, we checked for their presence in infected eyes [52]. We observed strong infiltration of microglial cells in USUV-infected eyes, mainly in close proximity to the RPE (Fig 7D). To see if inflammatory cell infiltration in eyes is associated or not with chemokine/cytokine overexpression, we measured mRNA expression of *CXCL10*, *CCL 5* and *IL6*, among the strongest inflammatory mediators overexpressed in previous tested organs (*i*.*e*., brain and spinal cord). All of these chemokines/cytokines were overexpressed after USUV infection whereas *IFNβ* and *TNFα* (tumor necrosis factor) levels remained unchanged (Fig 7E).

The RPE, the supporting tissue of the retina, consists of a monolayer of epithelial cells that contributes to the retinal-blood barrier and regulation of the immune response in the eye [53,54]. In these cells, viral replication could be associated with anti-viral responses and cytokine secretion, which could potentiate local inflammation. Human induced pluripotent stem cell (iPSC)-derived retinal pigment epithelium is morphologically and functionally characteristic of the RPE *in vivo* and thus represents a powerful model for studying pathophysiology of the RPE and its role during arboviral infection [55,56]. We infected human RPE, grown on transwell filters in compartmentalized chambers, with USUV at a MOI of 0.2. We observed viral production in apical and basolateral chambers over a period of 7 days by the TCID50 method (Fig 7F). Epithelium integrity was maintained upon infection and measures of the transepithelial resistance (TEER) of confluent RPE monolayers showed no significant variation after infection (S6 Fig). We performed RT-qPCR analysis targeting mainly genes involved in interferon response. This analysis disclosed upregulation of type 1 IFN (*IFNα* and *IFNβ*) and Interferon-Responsive Genes showing notably upregulation of many IFI (Interferon-inducible protein) (Fig 7G). Interestingly, CXCL10, CCL5 and IL6 were also found upregulated in human RPE infected by USUV, as was observed in the murine eyes as well as the brain and spinal cord.

Taken together, these data suggest that USUV replication in mouse eyes induces inflammation as in the CNS. Moreover, USUV infects human RPE and elicits a strong anti-viral response, potentially leading to immune-mediated responses.

## Discussion

The immune system initially is mobilized to defend the CNS from the invading pathogens, but sometimes can participate in devastating pathological reactions. Even with the great diversity of viruses that invade the CNS, many infections induce common pathogenic cascades such as the release of potentially detrimental immune mediators that can cause neurotoxicity. Therefore, despite the immune-privileged status of the CNS, inflammation can appear and induce neuronal damage in humans and animals. This inflammation can take place in several anatomical regions such as the meninges (meningitis), parenchyma (encephalitis), or spinal cord (myelitis), but also in multiple regions (meningoencephalitis, encephalomyelitis). Antiviral immune responses can also under certain conditions be an active contributor to these neurological disorders [57]. The symptoms and severity of the disorders thus depend on several factors, such as cell tropism, viral cytopathogenicity and the host immune response. One of our striking results is the massive inflammatory response in the brain and spinal cord of USUV-infected mice. Interestingly, the inflammatory signature is very similar in both organs. As part of this inflammatory signature, the chemokine CXCL10 plays a pivotal role in the attraction of leukocytes into the CNS, and was previously described to be upregulated after flavivirus infection like for WNV, which is closely related to USUV [41]. Notably this C-X-C chemokine was shown to be induced in several models of WNV brain and spinal cord infection as well as in WNV-infected blood donors [58–61]. CXCL10 can be secreted by numerous cell types, including leukocytes, monocytes, endothelial and epithelial cells, and is produced through the activation of IFN signaling or the lymphotoxin-β receptor LTβR via NF-κB [62]. This chemokine binds to the seven trans-membrane-spanning G protein-coupled receptor CXCR3, which regulates both chemotaxis and apoptosis of several immune cell types, and so can be either beneficial or detrimental to viral infection [42,63]. In response to USUV infection, this inflammatory context is associated with strong antiviral-response induction, as shown by up regulation of *IFNβ* and ISGs in the brain and spinal cord. These observations confirm previous studies showing induction of anti-viral response despite USUV replication, supporting the hypothesis that USUV does not possess mechanisms that interfere efficiently with IFN induction [45,64]. Mechanisms used by USUV to overcome this IFN response, while establishing a productive infection remains to be determined but the absence of efficient protective effect of type 1 IFN has been demonstrated for numerous other neurotropic flaviviruses [65–68]. In USUV-infected brains and spinal cords, we observed an increased expression of RIG-I, LGP2 (a positive regulator of RIG-I) and TLR3, PRRs known to be activated by viral dsRNA during viral replication [43]. Moreover, our results suggest an activation of the NLRP3 and AIM2 inflammasomes after USUV infection, as we detected overexpression of major components of these structures (i.e., *NLRP3*, *IL-1β*, *AIM2*, *CASP1*, *CASP8* and *PYCARD*) [40]. Inflammasome is a multiprotein complex organized in inflammasome sensor molecules like NLR, AIM2 or RIG- I connect to caspase 1 via ASC, which is an adaptor protein encoded by PYCARD that is common to all inflammasomes [69]. It plays an important role in the innate immune pathway and regulates at least two responses of the host: secretion of proinflammatory cytokines (IL-1β and IL-18) and induction of pyroptosis, a highly inflammatory form of programmed cell death that depends on the activation of at least one of the inflammatory caspases such as caspase-1 [69]. Several studies have demonstrated that some viruses have the ability to activate diverse inflammasomes, such as the NLRP3, AIM2, and RIG-I inflammasomes, which in turn contributes to mediate the host response to viral infection [40]. Moreover, inflammasome activation has been shown to be important for immune response and inflammation induced by several flaviviruses like DENV, ZIKV and WNV [70–72].

Studies of BBB infection are especially complicated due to a lack of pertinent models. Using an innovative human *in vitro* BBB model, recapitulating the main characteristics of the endothelial barrier, we showed that the human BBB is permissive to USUV replication. Despite the presence of proinflammatory cells and viral RNA in the brain, a significant decrease in barrier integrity was not observed at the time points assessed in this study contrary to what has been observed for WNV [73]. These results suggest that USUV directly infects vascular endothelial cells, which allow direct passage across the BBB into the CNS without compromising the BBB integrity. It is generally believed that BBB disruption is a determinant event preceding viral invasion; however, several studies suggest that neurotropic flaviviruses can reach the CNS by crossing the BBB without barrier breakdown, allowing free virus to spread in the CNS. Some viruses can directly infect brain endothelial cells, which allow direct passage across the BBB. Moreover, the CNS exhibits areas (such as the choroid plexus and circumventricular organs) that are not completely protected by the BBB and serve as entry points for viruses. Several neurotropic viruses can also reach the CNS as cell-associated particles through a mechanism called the “Trojan horse”, in which peripheral-infected leukocytes transmigrate through the endothelial cell layer and release the virus within the CNS parenchyma. Finally, some viruses infect and migrate through peripheral nerves by axonal transport [44]. Viruses can then infect resident cells in the brain such as neurons, microglia, astrocytes and pericytes. We previously showed that astrocytes and neurons are permissive to USUV infection and we display here *in vivo* and *in vitro* infection and recruitment/activation of microglia after USUV infection [30]. We detected enhanced secretion of chemokines by endothelial cells that is classically required to attract leukocytes to the brain. Among them, infected-leukocytes also could be recruited and release the virus within the CNS through the “Trojan horse” mechanism, as suggested for WNV [74]. Thus, global upregulation of inflammatory cytokines and chemokines in the brain could be responsible for leukocyte recruitment and participate in general immune cell CNS infiltration and inflammation-associated pathology.

Numerous infectious pathogens are known to directly affect the spinal cord or trigger autoimmune reactions, which may result in inflammation and permanent damage associated notably to acute flaccid paralysis or transverse myelitis [75]. Acute flaccid paralysis is characterized by lesions in the anterior horn cells of the spinal cord, with abnormalities noted on magnetic resonance imaging (MRI) predominantly found in the gray matter. Transverse myelitis is caused by inflammation of the spinal cord that may result from viral infections, abnormal immune reactions, or insufficient blood flow through the blood vessels. Inflammation can damage or destroy myelin and so interrupt communication between the nerves in the spinal cord and the rest of the body [75]. This can cause particularly muscle weakness, paralysis or sensory problems. Our results clearly showed that USUV targets the spinal cord, which is consistent with the motor defects that we observed. We also described an inflammation signature in the spinal cord with a cytokine profile that is very similar to what we observed in the brain.

Another striking effect of USUV infection is its capacity to infect mouse eyes and a human iPSC-derived RPE model. This last model is morphologically and functionally characteristic of the RPE *in vivo* presenting polarized pigmented cobblestone expressing characteristic RPE markers and tight junction proteins [42]. It thus represents a powerful tool to study the pathophysiology of the RPE. Anatomically and developmentally, the eyes are known as an extension of the CNS and are proposed to act as a reservoir of viral replication; moreover RPE are known to be targeted by numerous pathogens including arboviruses, such as DENV, ZIKV and WNV, leading to major ophthalmological damage [50,51,56,76]. Numerous studies have shown the critical role of immune cells in retinal disease, and neuroinflammation is an important aspect of many diseases of the eye. Like the brain the eye is also surrounded by an array of blood–ocular barriers that share structures and mechanisms with the CNS gating system. Our results showed disruption of both inner and outer retinal structure and a loss of photoreceptors layer in mouse eyes whereas USUV-infection does not seem to affect the integrity of the human ocular epithelium *in vitro*, but replication is associated with activation of inflammation through an immune-mediated reaction. The inflammatory signature in USUV-infected eyes is, at least partially, similar to that of the CNS. Interestingly, among the cytokines overexpressed in our ocular models, CXCL10 has already been shown to be implicated in various ocular disorders involving retinal degeneration and has been shown to be up-regulated in human RPE and in the eyes of mice infected systemically with ZIKV [56,63,77–79].

In conclusion, we showed that USUV infects multiple organs and cells of the CNS associated with drastic inflammation and various deleterious effects such as motor and ocular defects. Despite the fact that several neurological complications such as encephalitis or meningoencephalitis have been recently reported for USUV, this emergent virus has been much less studied than other related flaviviruses. Its recent spread in Europe, concomitant with that of the closely related WNV, must lead us to be vigilant and to better characterize the epidemiological and biological characteristics of USUV infection.

## Materials and methods

### USUV strains, production and cellular infection

Africa 2 strain of USUV (Rhône 2705/France/2015-KX601692), was provided by Anses (agence nationale de sécurité sanitaire de l’alimentation, de l’environnement et du travail) and was propagated three times on Vero cells (ATCC CCL-81). Viral stocks were prepared by infecting sub confluent Vero cells at a multiplicity of infection (MOI) of 0.01 in D-MEM medium (Thermoscientific) supplemented by 2% heat-inactivated fetal bovine serum (Sigma). Cell supernatant was collected 6 dpi and viral stock harvested after centrifugation at 300 *g* to remove cellular debris. Viral titers were determined by TCID50, which was calculated using the Spearman-Kärber method and were expressed as TCID50 per mL [80].

Cells at 60–70% confluence were rinsed once with phosphate-buffered saline (PBS), and USUV diluted to the required MOI were added to the cells in a low medium volume. Cells were incubated for 2 h at 37°C with permanent gentle agitation and then culture medium was added to each well, and cells were incubated at 37°C and 5% CO_2_. As a control, cells were incubated with the culture supernatant from Vero cells (mock condition).

### Mouse experiments

Pathogen-free *Ifnar*^*−/−*^ mice [81] kindly provided by Dr. Gilles Uzé were backcrossed onto a C57BL/6 background. Swiss mice were purchased from Janvier Laboratories (Saint-Berthevin Cedex, France). Mice were infected at E.C.E. (Etablissement Confiné d’Expérimentation), a level 3 animal facility of the University of Montpellier.

Groups of 8 to 12-week-old males for *Ifnar*^*−/−*^ mice and of 6 days for studies in neonatal Swiss animals were inoculated intraperitoneally with 10^4^ TCID50/mouse of USUV. USUV-infected mice and control mice were euthanized by cervical dislocation or with a lethal dose of pentobarbital (Sigma-Aldrich Darmstadt, Germany) at indicated dpi depending on the experimental design. At 3 and 6 dpi three mock- and USUV-infected mice were i.p. injected with 0.5% Evans blue solution (200 μL per mouse) for 6 hours and euthanized after PBS intra-cardiac perfusion. Organs and tissues (urine, blood, liver, spleen, kidney, muscle, bladder, eye, brain, spinal cord, sciatic nerve) were snap frozen with liquid nitrogen for viral burden and Elisa analysis or collected after PBS intra-cardiac perfusion, fixed in 4% paraformaldehyde (PFA) and cut using a microtome (3 μm sections) at the RHEM facilities (Montpellier). Animals were sacrificed if they present deterioration of body condition score and drastic weight loss (more than 20%).

### Ethics statement

Mice were bred and maintained according to the French Ministry of Agriculture and European institutional guidelines (appendix A STE n°123). Experiments were performed according to national regulations and approved by the regional ethics committee of Languedoc-Roussillon (Comité Régional d'Ethique sur l'Expérimentation Animale- Languedoc-Roussillon), France (approval N° 6773–201609161356607).

For human BBB model all adult subjects provided written informed consent, and a parent or guardian of any child participant provided informed consent on the child’s behalf, in compliance with the French legislation. The protocol was approved by the French Ministry of Higher Education and Research (CODECOH Number DC2011-1321).

### Motor tests

#### Footprint analysis

Mouse hindpaws were dipped in nontoxic water-based paints. Mice were then allowed to walk down an enclosed runway lined with white paper. Three trials were performed for each mouse. Two to four steps from the middle portion of each run were measured for hind-stride length. Hindlimb foot angle is used to determine gait abnormalities. It is measured by drawing a line from the mid-heel through the middle (longest) digit. At least nine steps were measured for each mouse. Mean values were used for statistical analysis.

#### Inverted screen

The subject was placed on a grid screen. The grid was waved lightly in the air, then inverted 60 cm over a cage with soft bedding material. Mice were tested only one time with a 60-s maximum cutoff, and the latency to fall was recorded.

#### Histology and immunostainings

Samples were collected and fixed 24h in neutral buffered formalin 10%. Brain and eyes were dehydrated and embedded in paraffin for brain and eyes. Spinal cords were removed, post-fixed and embedded in Tissue-Tek OCT Compound (Zoeterwoude, the Netherlands); sections (12 μm) of lumbar region were stained with CD45 (Bioscience, 14–0451, 1∶500) and then mounted in Eukitt. Paraffin-embedded tissue was cut into 3-μm-thick sections, mounted on slides, then dried at 37°C overnight. Tissue sections were stained with HES with HMS 740 autostainer (MM France) for preliminary analyses. Immunohistochemical staining was performed on the Discovery Ultra Automated IHC staining system using the Ventana DAB Map detection kit. Following deparaffination with Discovery EZ Prep solution at 75°C for 24 minutes, antigen retrieval was performed at 95–100°C for 24 or 32 minutes using Discovery CC1 buffer for PAX5 and CD3e stainings respectively for 15 minutes using Discovery RiboCC buffer for CD45 staining, and 24 minutes using Discovery CC2 buffer for Cleaved caspase 3 staining. Endogenous peroxidase was blocked with Discovery Inhibitor CM for 8 minutes at 37°C. The slides were incubated after rinsing at 37°C for 60 minutes with a goat anti-PAX5 antibody (Santa cruz, sc1974, 1∶125), a goat anti-CD3e antibody (Santa cruz, sc1127, 1∶2000), a rabbit anti-Cleaved caspase 3 antibody (Cell signaling, 9671, 1:4000) or a rat anti-CD45 antibody (Bioscience, 14–0451, 1∶500). Signal enhancement was performed using the Discovery DAB Goat OmniMap Kit for CD3e and PAX5 stainings, Discovery DAB Rabbit OmniMap Kit for Cleaved Caspase 3 staining and rabbit anti-rat IgG (H+L) as the secondary antibody (Thermo Scientific, 31219) and the Discovery DAB Rabbit OmniMap Kit for CD45 staining. For viral antigen immunostaining, antigen retrieval was performed for 4 minutes at 37°C with Protease 1 solution from Ventana which is an endopeptidase (alkaline protease) of the serine protease family. Endogenous peroxidase was blocked with Discovery Inhibitor CM for 8 minutes at 37°C. The slides were incubated after rinsing at 37°C for 32 minutes with a mouse anti-flavivirus group antigen monoclonal antibody (Millipore, MAB10216, 1∶800 in Dako antibody diluent with background reducing components). Signal enhancement was performed using Rabbit monoclonal to mouse IgG1 + IgG2a + IgG3 as the secondary antibody (Abcam, ab133469, 1/8000) and the Discovery DAB Rabbit HQ Kit. Slides were then counterstained for 8 minutes and manually dehydrated before coverslips were added. Slides were treated with a Hamamatsu NanoZoomer 2.0-HT scanner by MRI platform and images were visualized with the NDP.view 1.2.47 software. Positive cells were counting using QuPath bioimage analysis software.

### Immunofluorescence assays

For indirect immunofluorescence, cells were fixed with 4% PFA and permeabilized with 0.1% Triton X-100/PBS for 5 min at room temperature (RT), followed by a blocking step with 2% bovine serum albumin (BSA) and 10% horse serum for 30 min to 1h at RT. Primary and secondary antibodies were diluted in blocking solution and incubated sequentially for 1h at RT. Samples were then mounted with fluorescent mounting medium (Prolongold, Thermofischer) with Hoescht (Sigma) and imaged by confocal microscopy using the Zeiss SP85 confocal microscope, with 40× or 63× 1.4 NA Plan Apochromat oil-immersion objectives.

### ELISA (Enzyme-Linked Immunosorbent Assay)

ELISA assays for human CXCL10, IL6, CCL5 were performed in mock- and USUV-infected supernatants from hBLECs at various dpi according to the manufacturer’s instructions (R&D systems). Reading were done on spectrophotometer (ThermoFischer Scientifics) and data were analysed using the xPONENT program. Mean concentrations (pg/mL) of cytokines were all superior to the detection limits, defined as the mean background value plus standard deviations (stddev).

### Measurement of viral burden *in vivo*

Organs were weighed and homogenized with zirconia beads in a Fastprep 24 apparatus (MP Biomedicals) in 250 or 500 μL PBS and stored at −80°C. RNA was extracted using the RNeasy Mini Kit (Qiagen). Blood and urine RNAs were extracted at 4, 6, 9, 12 and 16 dpi from 100 μL of samples, with the EZ1 apparatus running the EZ1 DSP virus kit (Qiagen). Viral RNA levels were measured by a one-step quantitative reverse transcriptase PCR assay (RT-qPCR) on the Light Cycler 480 (Roche) with primers, probe and cycling conditions previously described [37]. Viral burden was expressed on a log10 scale as TCID50 equivalents per gram after comparison with a standard curve produced using serial 10-fold dilutions of USUV with known viral titers.

### RT-qPCR assays

USUV-or mock-infected cells (RPE cultured in 24-well plates) or mouse organs homogenized (brain and spinal cord) were harvested in RLT buffer (Qiagen) and total RNA was extracted using RNeasy mini-kit (Qiagen). Complementary DNA was synthesized using Omniscript reverse transcriptase (Life Technologies). RT^2^ Profiler PCR Array Human Interferons & Receptors (RPE, # PAHS 064Z, 96 well format, Qiagen, 84 genes analyzed) or for Mouse Inflammatory Cytokines & Receptors RT (#PAMM-011Z, 96 well format, Qiagen, 84 genes analyzed) as well as single transcript levels analysis were performed using the LC480 real time PCR instrument (Roche) and the Light Cycler 480 SYBR Green I master Mix (Roche). Volumes of mix, cDNA, RNAse-free water, and cycling conditions were determined according to the manufacturer's instructions. Gene expression was normalized to that of the housekeeping gene HPRT. Genes without interpretable amplification curves were excluded from the analysis.

### Immunoblotting

Samples were lysed by boiling in SDS sample buffer, sonicated, and complemented with dithiothreitol (DTT). Protein concentrations were measured by a bicinchoninic acid (BCA) protein assay kit (Pierce, MA, USA). Equal amounts of protein from total cell lysates (10 μg) were loaded on SDS-PAGE gels and transferred onto nitrocellulose membranes. The membranes were blocked and incubated overnight at 4°C with primary antibodies and then incubated with horseradish peroxidase (HRP)-conjugated secondary antibodies (Amersham) for 1 h, bands were visualized by ChemiDoc XRS plus (Biorad Laboratories Hercules, CA).

### *In vitro* human BBB model

The *in vitro* human BBB model consists in CD34^+^ blood cord-derived endothelial cells (CD34^+^-EC) that have been further differentiated and cultured in transwell filters (Costar) on top of bovine pericytes in 12-well plates as previously described [46]. Briefly, once plated, CD34^+^-EC and pericytes were cultured for 5–6 days with medium change (Endothelium Cell Medium (ECM) supplemented with 5% Fetal Calf Serum (ECM-5)) every 2 days. Then, endothelial permeability (Pe) was tested by measuring Lucifer Yellow (LY, 20 μM; Life Technologies) transendothelial crossing using established protocols [46,82]. Pe was measured after 1 hour of LY transport by calculating the concentration-independent parameter as previously published [82]. The fluorescence detection was performed using a Tecan apparatus with excitation/emission wavelength (nm) settings of 432/538. Infection experiments were carried when the barrier was impermeable (*i*.*e*. with a Pe ≤ to 1,5x10^-3^ cm/min). USUV was added at the MOI of 0,1 in 200 μL of ECM-5 for 2h on an orbital shaker. 300 μL of ECM-5 were then added and the inoculum removed. Infections were then carried for 4, 7 or 10 days with medium change at the same time. For each experiment, triplicates were used.

### iPSC-derived RPE generation and culture

Human iPSCs were generated from wild type BJ fibroblasts (ATCC CRL2522) as previously described [83]. iPSCs were cultured in Knockout DMEM medium (ThermoFischer Scientific) supplemented with 20% KO serum replacement (ThermoFischer Scientific), 1% GlutaMAX (ThermoFischer Scientific), 1% non-essential amino acids (ThermoFischer Scientific), 0.1% β-mercaptoethanol (ThermoFischer Scientific) and 1% penicillin-streptomycin (ThermoFischer Scientific). Six weeks later, pigmented foci were manually dissected, pooled, dissociated with 0.25% trypsin, filtered through a 40-μm filter and seeded at a density of ~3x10^4^ cells per 0.32 cm^2^ on a 1/30 dilution Corning Matrigel HESC-qualified matrix (Dominique Dutscher). Experiments were performed on iPSC-derived RPE at P3. USUV was added at the MOI of 0,2 in 200 μL of medium for 2h on an orbital shaker. 300 μL of new medium were then added and the inoculum removed.

### Transepithelial resistance (TEER) measurements

The iPSC-derived RPE was cultured on Matrigel-coated, clear BD Falcon cell culture inserts with high density 0.4 μM pores (Dominique Dutscher) in 24-well plates at P3. The TEER was measured using the Epithelial Volt/Ohm Meter EVOM2 (World Precision Instruments, Hertfordshire, U.K.) according to the manufacturer’s instructions as previously described [56]. Electrodes were sterilized in 70% ethanol for 5 min, rinsed and equilibrated in media, then placed in the chambers with the longer electrode vertically touching the bottom of the dish in the lower chamber and the shorter electrode in the upper chamber without touching the cell layer. To calculate the final TEER values (Ohms.cm^2^), the background measurement of a Matrigel-coated insert without cells was subtracted from the reading and the value multiplied by the growth surface area.

### Material

Antibodies used in this study are: mouse anti-pan-flavivirus (clone 4G2, MAB10216 Millipore), rabbit anti-β-catenin, (clone E247, Abcam), goat anti-PAX5 antibody (Santa cruz, sc1974), goat anti-CD3e antibody (Santa cruz, sc1127), rabbit anti-Cleaved caspase 3 antibody (Cell signaling, 9671), rat anti-CD45 antibody (Bioscience, 14–0451), rabbit monoclonal anti-iba1 (Abcam, ab 178847), actinGreen (R37110, ThermoFischer scientific), rabbit polyclonal anti-CXCL10 (Abcam, ab 9938), mouse monoclonal anti-GAPDH (Abcam, ab 8245).

### Statistical analyses

For quantitative analyses, a minimum of three independent experiments were performed. Student’s *t*-test and the Mann-Whitney test were performed to analyze unpaired data (* p < 0.05, ** p <0.01). Data were analyzed with GraphPad Prism software.

## Supporting information

S1 Fig Histological sections of neonatal brain control mice. Immunohistochemical staining of B-cells (PAX 5), T-cells (CD3) and macrophages (F4/F80).
(TIF)

S2 Fig ELISA analyses of CXCL10, IL6 and CCL5 concentrations in the blood of control- or USUV-infected mice at 6 dpi for Swiss mice and at 3 dpi for IFNAR mice. (TIF)

S3 Fig *p*-values of RT-qPCR analyses from PCR array. *p*-values of RT-qPCR analyses of the specific inflammatory cytokines and receptors PCR array from USUV-infected neonatal versus control brain (A) and (B) spinal cord at 6 dpi.
(TIF)

S4 Fig *p*-values of RT-qPCR analyses from PCR array. *p*-values of RT-qPCR analyses of the specific interferons and receptors PCR array from USUV-infected RPE versus control (mock) RPE at 2 dpi.
(TIF)

S5 Fig Immunofluorescence caspase 3 staining showing no apoptosis in USUV-infected eyes. (TIF)

S6 Fig (A) iPSC-derived RPE were grown to confluence at passage 3 and showed classical pigmented cobblestone structure by light microscopy in control and infected cells at day 7. (B) Measures of RPE transepithelial resistance (TER) were performed using an epithelial volt-ohm meter before and after USUV infection at a MOI of 0.1. No significant variation was found in USUV-infected RPE versus control cells. Results are expressed as mean ± SEM, n = 3 independent experiments.
(TIF)
