# Supplementary figures and images for: Study of Usutu virus neuropathogenicity in mice and human cellular models
Source: PLoS Negl Trop Dis. 2020 Apr 23;14(4):e0008223. doi: 10.1371/journal.pntd.0008223 (PMC7179837; doi:10.1371/journal.pntd.0008223)

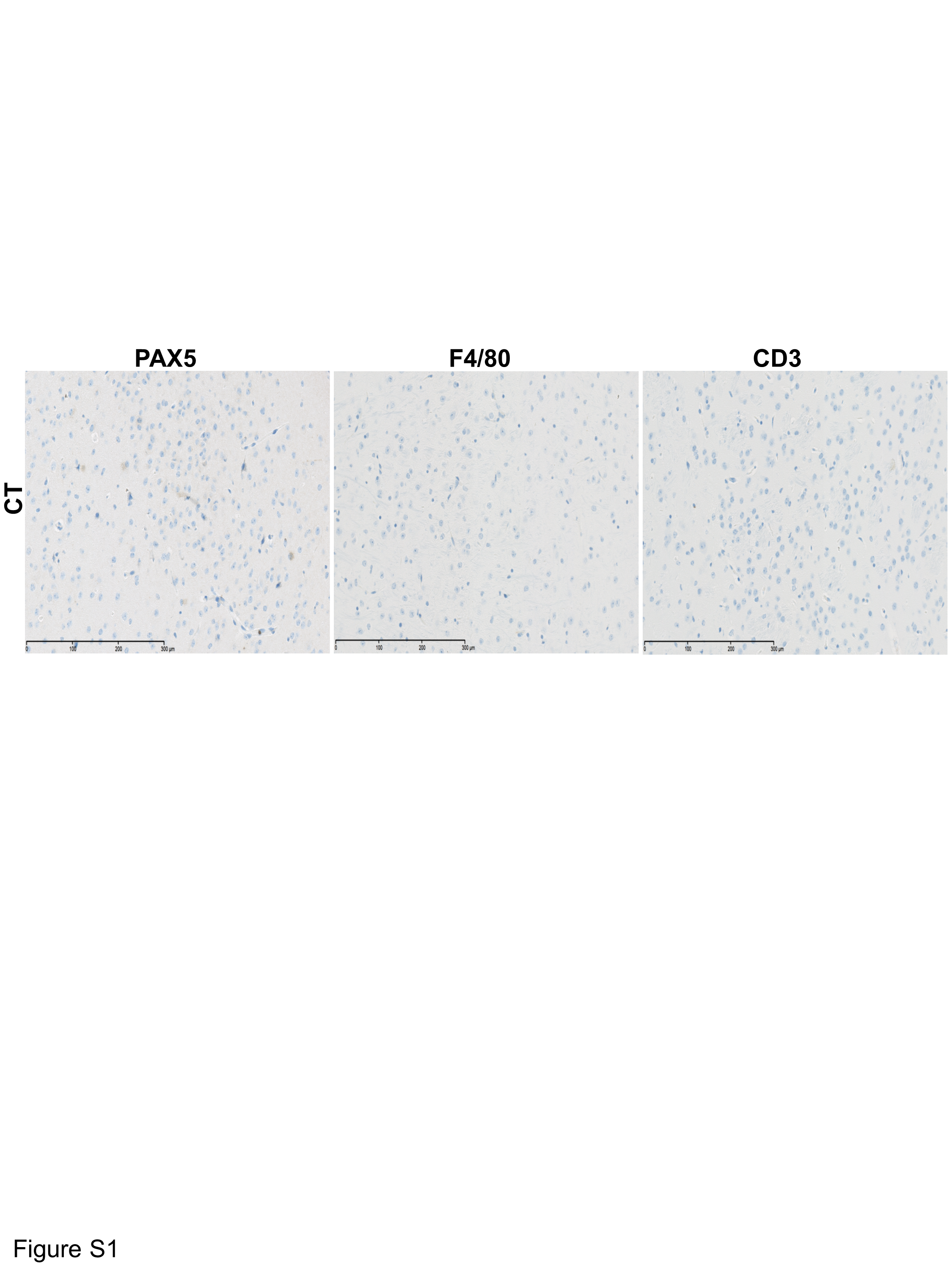

Supplement: S1 Fig — Immunohistochemical staining of B-cells (PAX 5), T-cells (CD3) and macrophages (F4/F80). (TIF) [file pntd.0008223.s001.tif]

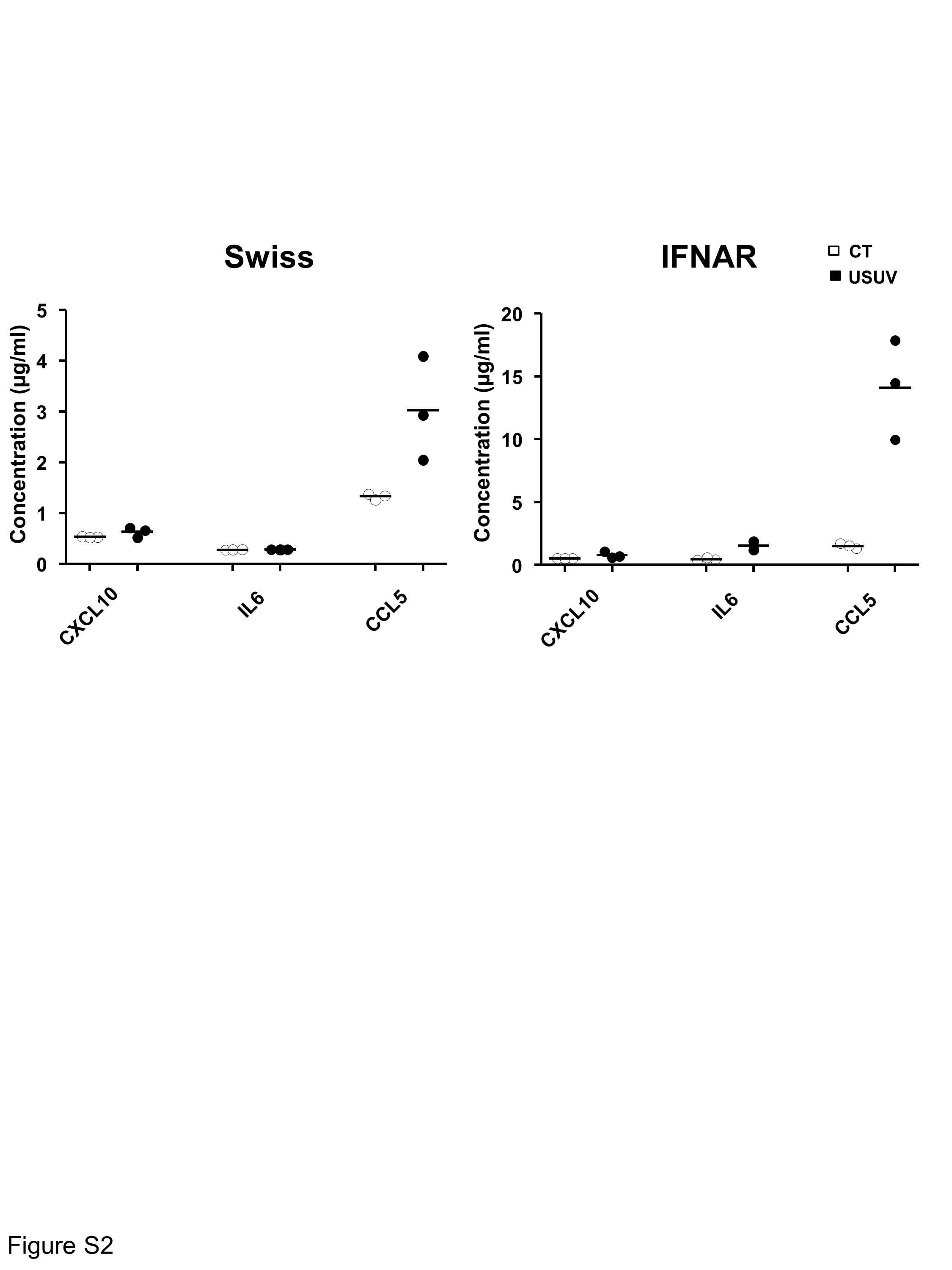

Supplement: S2 Fig — (TIF) [file pntd.0008223.s002.tif]

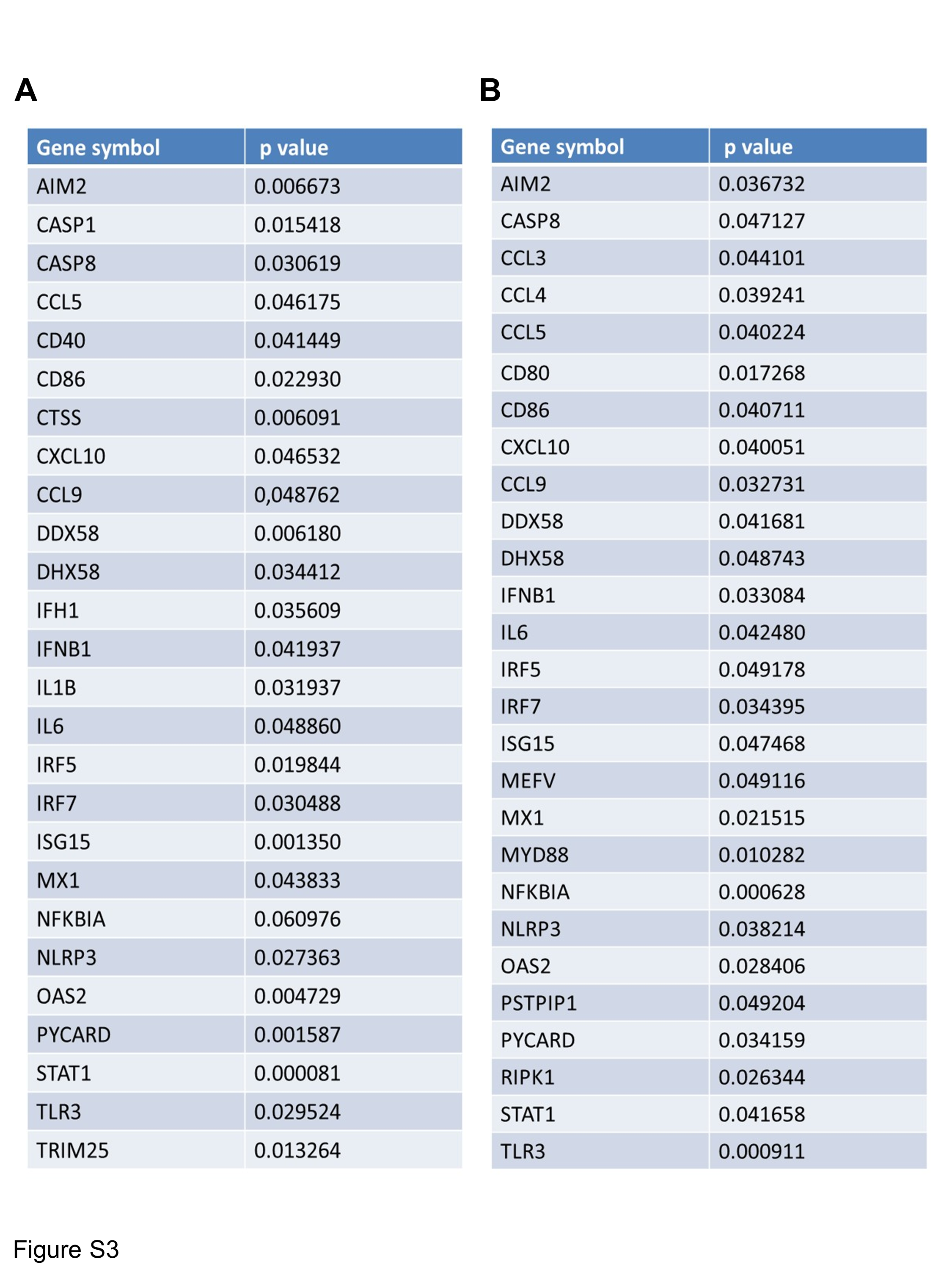

Supplement: S3 Fig — p-values of RT-qPCR analyses of the specific inflammatory cytokines and receptors PCR array from USUV-infected neonatal versus control brain (A) and (B) spinal cord at 6 dpi. (TIF) [file pntd.0008223.s003.tif]

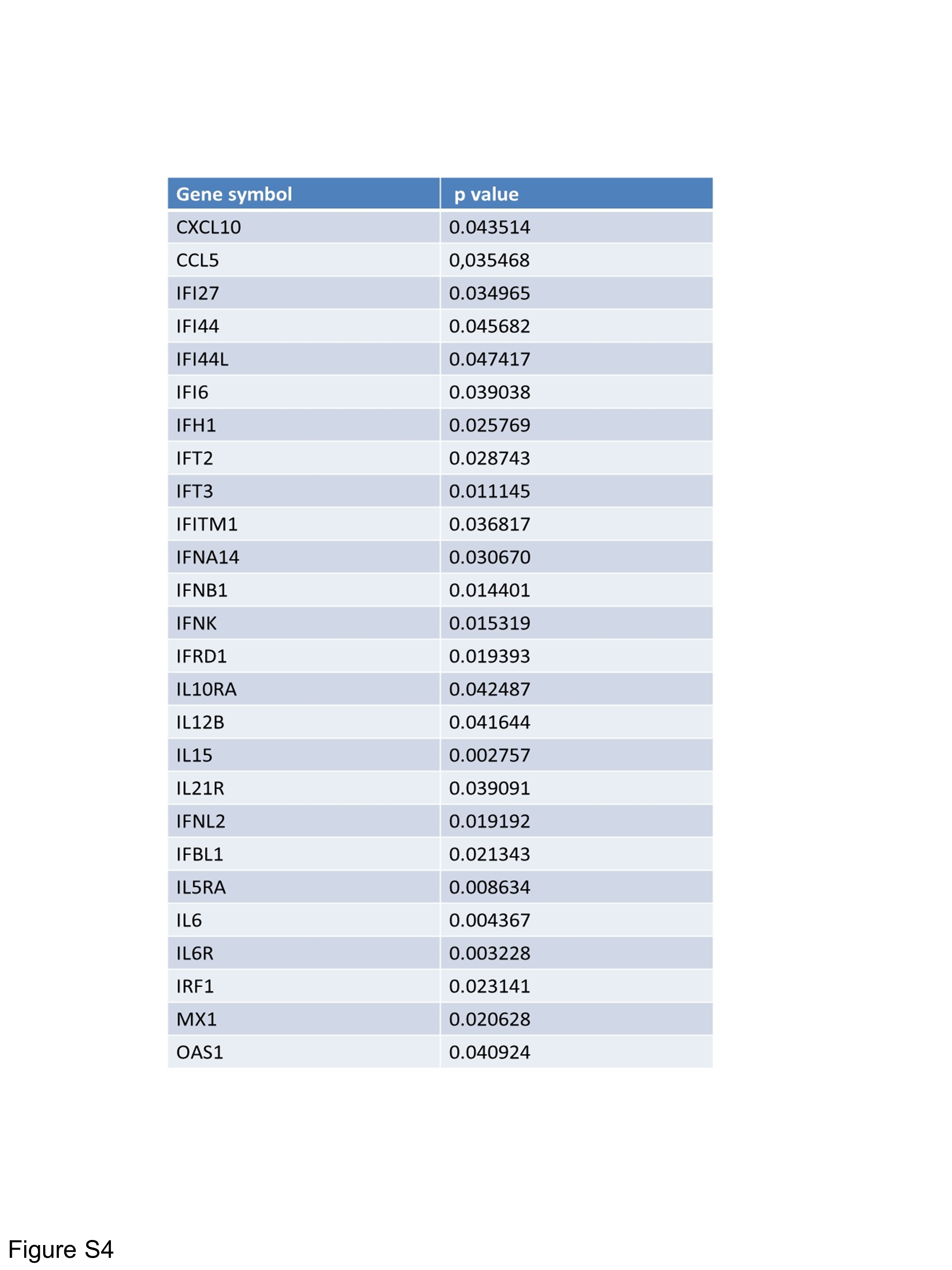

Supplement: S4 Fig — p-values of RT-qPCR analyses of the specific interferons and receptors PCR array from USUV-infected RPE versus control (mock) RPE at 2 dpi. (TIF) [file pntd.0008223.s004.tif]

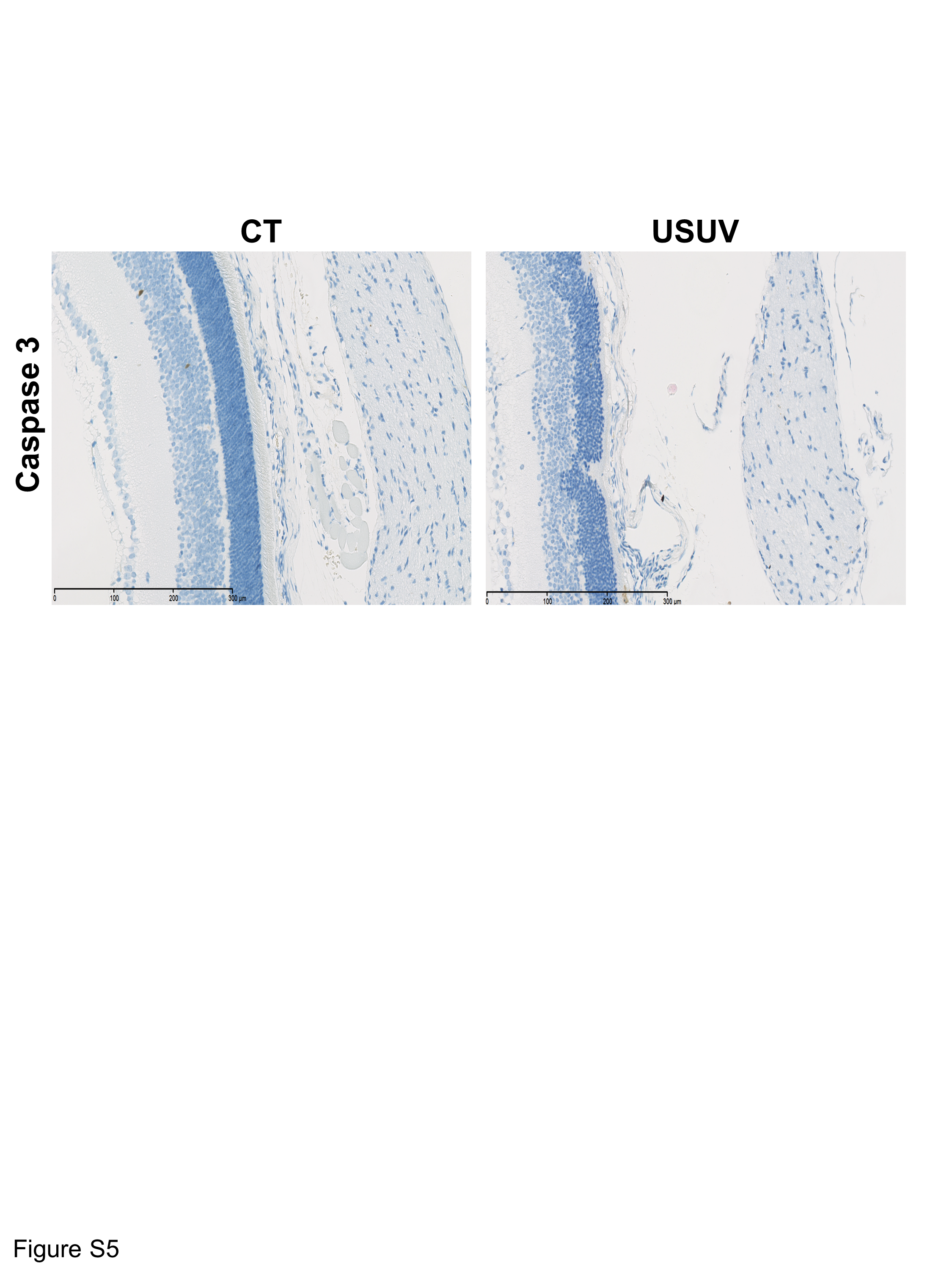

Supplement: S5 Fig — (TIF) [file pntd.0008223.s005.tif]

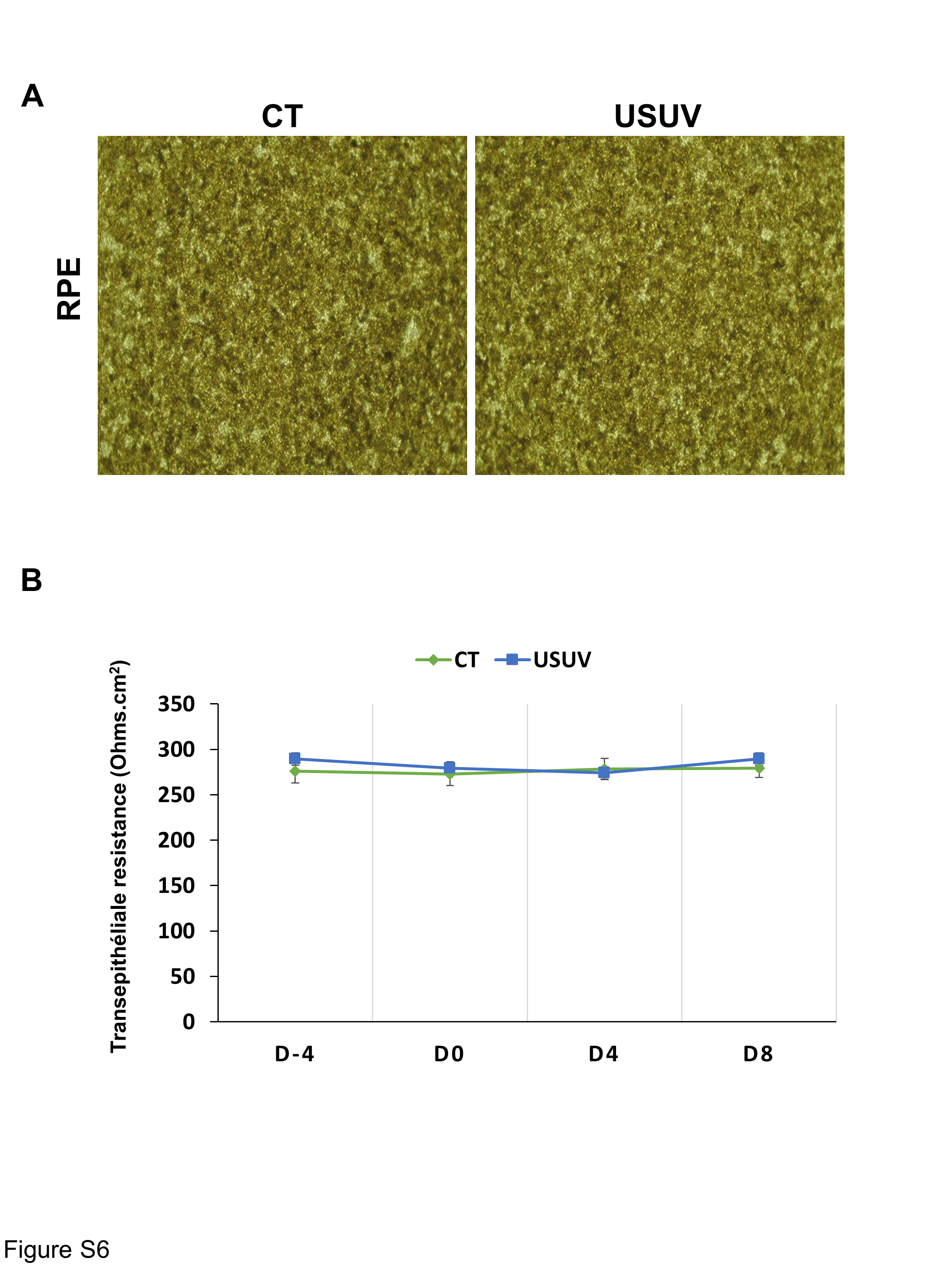

Supplement: S6 Fig — (A) iPSC-derived RPE were grown to confluence at passage 3 and showed classical pigmented cobblestone structure by light microscopy in control and infected cells at day 7. (B) Measures of RPE transepithelial resistance (TER) were performed using an epithelial volt-ohm meter before and after USUV infection at a MOI of 0.1. No significant variation was found in USUV-infected RPE versus control cells. Results are expressed as mean ± SEM, n = 3 independent experiments. (TIF) [file pntd.0008223.s006.tif]
